# Leistenrezidive nach standardisierter Crossektomie und Stripping der Vena saphena magna – 5‐Jahres‐Ergebnisse der LaVaCro‐Studie

**DOI:** 10.1111/ddg.70012x

**Published:** 2026-10-05

**Authors:** Thomas Hummel, Georgios Papapostolou, Margit Broermann, Christoph Dresler, Ingo Flessenkämper, Michael Jünger, Dietmar Stenger, Dominic Mühlberger, Yazad Sidhwa, Thomas Falkenstein, Markus Stücker, Achim Mumme

**Affiliations:** ^1^ Interdisziplinäres Venenzentrum der Kliniken für Dermatologie und Gefäßchirurgie Katholisches Klinikum der Ruhr‐Universität Bochum Bochum Deutschland; ^2^ Venenzentrum der Artemed Fachklinik München Deutschland; ^3^ Venenzentrum Northeim Northeim Deutschland; ^4^ Gefäßzentrum Berlin Südwest HELIOS Klinikum Emil von Behring Berlin Deutschland; ^5^ Klinik und Poliklinik für Hautkrankheiten Universitätsmedizin Greifswald Greifswald Deutschland; ^6^ Venenzentrum Saarlouis Saarlouis Deutschland

**Keywords:** *High ligation*, Leistenrezidiv, sapheno‐femorale Crosse, Varizen, Vena saphena magna, Great saphenous vein, high ligation, saphenofemoral junction, groin recurrence, varicose veins

## Abstract

**Hintergrund:**

Rezidive im Bereich der sapheno‐femoralen Crosse (SFJ) nehmen einen erheblichen Anteil der täglichen phlebologischen Praxis ein. Die Langzeitergebnisse nach *High Ligation* und *Stripping* (HL/S) der Vena saphena magna (VSM) sind bislang heterogen und teilweise widersprüchlich. Diese prospektive, multizentrische Studie wurde konzipiert, um die Ergebnisse nach standardisierter HL/S mit besonderem Fokus auf Leistenrezidive zu untersuchen.

**Patienten und Methodik:**

Es wurde eine standardisierte und einheitliche HL/S der VSM durchgeführt, einschließlich des Verschlusses der Fascia cribrosa. Der primäre Endpunkt war das Auftreten eines Leistenrezidivs, erfasst durch klinische Untersuchung und Duplexsonographie. Sekundäre Zielparameter waren Rezidive an anderen Lokalisationen.

**Ergebnisse:**

Insgesamt wurden 405 Patienten (519 Beine) aus 6 deutschen Venenzentren in die Studie eingeschlossen. 225 Patienten (287 Beine; 55,56 %) standen für die Fünf‐Jahres‐Nachuntersuchung zur Verfügung. Bei 21 Beinen (7,32 %) trat eine klinisch manifeste Varikose auf. Leistenrezidive wurden in 5 Fällen (1,74 %) festgestellt, von denen lediglich zwei klinisch relevant waren. Die Mehrzahl der klinisch erkennbaren Varizen war auf Perforansinsuffizienzen zurückzuführen.

**Schlussfolgerungen:**

Die standardisierte Operationstechnik der HL/S – einschließlich der Ligatur kleiner Seitenäste der Vena femoralis communis in unmittelbarer Nähe der SFJ sowie des Verschlusses der Fascia cribrosa als anatomische Barriere – zeigt auch nach 5 Jahren exzellente Ergebnisse.

## EINLEITUNG

Sapheno‐femorale Rezidive nach Crossektomie und Stripping der Vena saphena magna (VSM) besitzen eine hohe sozialmedizinische und ökonomische Bedeutung. Nach einer Untersuchung von Mumme et al. belaufen sich die jährlichen Kosten für die Behandlung solcher Rezidive auf über 50 Millionen Euro.^1^ Auf Grundlage der derzeit verfügbaren Studien ist die tatsächliche phlebologische Versorgungssituation bei Rezidiven jedoch nur schwer zu erfassen.

Aus hämodynamischer Sicht sind insbesondere Leistenrezidive von besonderer Relevanz. Aufgrund der anatomisch hohen Lage des Insuffizienzpunktes ist bei distaler gelegenen Varizen mit potenziell höheren venösen Drücken zu rechnen als bei weiter peripher lokalisierten Insuffizienzen. Dies könnte in Zukunft zu erhöhter Inzidenz symptomatischer Rezidive führen. Schul et al. konnten beispielsweise zeigen, dass eine Seitenastvarikose mit einem Insuffizienzpunkt auf identischer anatomischer Höhe dieselbe Morbidität aufweist wie eine Stammvarikose.^2^

In der deutschen Leistenrezidiv‐Studie wurde die Ursache von Leistenrezidiven histopathologisch bei 427 Patientinnen und Patienten untersucht.^3^, ^4^ Mit 65,3 % war ein Reflux aus einem verbliebenen Stumpf der VSM, der bei der Primäroperation auf Höhe der tiefen Vene nicht vollständig entfernt worden war, die häufigste Ursache. In 16 % der Fälle war eine Neovaskularisation ursächlich, die zu einer erneuten Verbindung zwischen dem tiefen und dem oberflächlichen Venensystem auf Höhe der sapheno‐femoralen Crosse (saphenofemoral junction, SFJ) führte.

Die Inzidenz sapheno‐femoraler Rezidive nach technisch korrekter Crossektomie ist bislang nicht eindeutig geklärt. Die Bewertung der Rezidivraten wird durch die lange Latenzzeit zwischen Operation und klinischem Auftreten erschwert. Nach Ergebnissen der deutschen Leistenrezidiv‐Studie werden sapheno‐femorale Rezidive im Mittel erst nach 7 bis 8 Jahren klinisch manifest.^3^, ^4^

Klinischen Rezidiven geht in der Regel ein pathologischer Reflux aus der SFJ voraus, der duplexsonographisch nachweisbar ist. Ein neu auftretender sapheno‐femoraler Reflux kann somit als Surrogatparameter für die Entwicklung eines klinischen Rezidivs dienen.^5^ In mehreren randomisierten Studien, die Langzeitergebnisse verschiedener Therapieoptionen bei Stamminsuffizienz der VSM untersuchten, wurde der duplexsonographische Nachweis eines pathologischen Refluxes aus der SFJ als primärer Endpunkt definiert.

Mittlerweile liegen mehrere Studien mit einer Nachbeobachtungsdauer von 5 Jahren vor.^6^, ^7^, ^8^, ^9^, ^10^, ^11^, ^12^, ^13^, ^14^, ^15^, ^16^, ^17^ Die berichteten Rezidivraten schwanken dabei zwischen 3,8 % und 36 %. Unterschiede in der Operationstechnik dürften wesentlich zur großen Spannbreite der Ergebnisse beigetragen haben, was die Interpretation der Daten erschwert.

Ziel der vorliegenden Untersuchung war es, die Rezidivraten nach technisch korrekter Crossektomie unter Anwendung einer streng standardisierten Operationstechnik in einer großen Patientenkohorte zu analysieren. Zur Erreichung dieses Ziels wurde eine prospektive, multizentrische Beobachtungsstudie initiiert – die LaVaCro‐Studie („Langzeitergebnisse der Varizenoperation mit Crossektomie und Stripping der Vena saphena magna“).^18^ Die vorliegende Analyse präsentiert die Fünf‐Jahres‐Ergebnisse dieser Studie.

### Material und Methodik

Voraussetzung für die Teilnahme der Venenzentren an der Studie war eine hohe Operationsfrequenz mit mindestens 500 *High‐Ligation*‐ und *Stripping*‐Eingriffen (HL/S) der Vena saphena magna (VSM) pro Jahr, um die chirurgische Erfahrung und Qualifikation der Operateure sicherzustellen. Darüber hinaus wurde für die Einbeziehung eines Zentrums in die Auswertung der Langzeitergebnisse eine Fünf‐Jahres‐Nachuntersuchungsrate von mindestens 30 % gefordert, um potenzielle Verzerrungen durch hohe Ausfallraten von Patientinnen und Patienten oder teilnehmenden Zentren zu vermeiden.

Die Operationen wurden in einer standardisierten, einheitlichen Technik gemäß der zuvor beschriebenen Methodik durchgeführt.^18^ Das standardisierte Vorgehen umfasste die Präparation der VSM, der SFJ sowie des angrenzenden Abschnitts der Vena femoralis communis (jeweils 0,5 cm ober‐ und unterhalb der SFJ) über einen kleinen Leistenzugang. Alle kleinen Äste des oberflächlichen Venenkonfluenzsystems sowie Zuflüsse, die direkt in die Vena femoralis communis münden, wurden ligiert. Zudem wurde die VSM mittels nichtresorbierbarem, monofilem Nahtmaterial unmittelbar auf Höhe der Einmündung in die Vena femoralis communis ligiert (*high ligation*). Anschließend erfolgte eine bipolare Koagulation des freiliegenden Endothels des ligierten VSM‐Stumpfes, um das Risiko einer Neovaskularisation zu reduzieren. Ein weiteres Grundprinzip der Operation war der Verschluss der Fossa ovalis durch Vernähen der Fascia cribrosa zur Schaffung einer natürlichen Barriere zwischen oberflächlichem und tiefem Kompartiment nach Schnyder et al.^19^ Danach wurden das invaginierende Stripping der VSM und der Wundverschluss der Leiste durchgeführt. Ergänzend erfolgten Mini‐Phlebektomien an Oberschenkel und Unterschenkel entsprechend dem Verlauf variköser Seitenäste.

Patienten mit einer symptomatischen Insuffizienz der VSM wurden im Rahmen der klinischen Routine in die Studie eingeschlossen. Der Reflux in der VSM wurde gemäß der Klassifikation nach Hach^20^ in vier Stadien eingeteilt (Hach 1: Reflux auf die SFJ begrenzt; Hach 2: Reflux von der SFJ bis zum Oberschenkel; Hach 3: Reflux von der SFJ bis zur Wade; Hach 4: Reflux von der SFJ bis zum Knöchel). Eingeschlossen wurden nur Patienten mit Hach Stadium 2–4. Zusätzlich wurde das C‐Stadium der CEAP‐Klassifikation dokumentiert.^21^

Die Studienmethodik wurde bereits ausführlich beschrieben.^18^ Patienten mit einem auf die SFJ begrenzten Reflux (Hach 1) oder fehlender Einverständniserklärung wurden von der Studie ausgeschlossen.

Nach 5 Jahren wurden die Patienten zur Nachuntersuchung eingeladen. Die Duplexsonographie umfasste folgende Venenabschnitte: Vena femoralis communis mit besonderem Fokus auf die ehemalige SFJ, Vena femoralis, Vena profunda femoris, Vena poplitea, Vena tibialis anterior et posterior, Venae peroneae sowie Perforansvenen des Oberschenkels und Unterschenkels.

Der primäre Endpunkt der Analyse war die Rezidivhäufigkeit im Bereich der SFJ nach HL/S der VSM. Sekundäre Endpunkte umfassten weitere klinisch relevante Rezidivraten, duplexsonographisch nachweisbare Rezidive an Oberschenkel und Unterschenkel sowie operationsbedingte Komplikationen oder andere Nebenwirkungen.

Ein Leistenrezidiv wurde definiert als ein pathologischer Reflux, der aus der Vena femoralis communis in das oberflächliche Venensystem übertritt. Ein Reflux mit einer Dauer von mehr als 0,5 Sekunden wurde als pathologisch gewertet.^22^ Venöse Refluxe ohne Verbindung zur Vena femoralis communis wurden nicht als Leistenrezidiv gewertet. Rezidive wurden als klinisch und hämodynamisch relevant betrachtet, wenn der Durchmesser der insuffizienten Vene allein oder in Summe über 5 mm betrug. Darüber hinaus wurde bei der Nachuntersuchung die CEAP‐Klassifikation erneut erhoben und eine Duplexsonographie der Leiste und des Beins durchgeführt, um den venösen Status der oberflächlichen und tiefen Kompartimente zu beurteilen und neu aufgetretene pathologische Refluxe zu identifizieren. Klinisch manifeste Varizenrezidive wurden anhand der REVAS‐Klassifikation (*Recurrent Varices After Surgery*) beurteilt.^23^, ^24^

Die Studie wurde in Übereinstimmung mit der Deklaration von Helsinki durchgeführt. Das Studienprotokoll wurde von der Ethikkommission der Medizinischen Fakultät der Ruhr‐Universität Bochum genehmigt (Registrierungsnummer: 3690‐10). Alle Patienten gaben eine schriftliche Einverständniserklärung, und die Datenerhebung erfolgte vollständig anonymisiert. Die statistische Auswertung erfolgte mit Microsoft Excel 2010 (deskriptive Statistik und Student's t‐Test); die Daten werden als Häufigkeitsverteilungen und Prozentangaben dargestellt. Ein p‐Wert < 0,05 wurde als statistisch signifikant gewertet.

## ERGEBNISSE

Die LaVaCro‐Studie wurde ursprünglich mit zwölf deutschen Venenzentren begonnen.^18^ Sechs dieser Zentren erreichten jedoch nicht die geforderte Nachuntersuchungsrate von 30 %. Daher konnten nur sechs Zentren, die diese Voraussetzung erfüllten, in die Auswertung der Langzeitergebnisse einbezogen werden, um eine wesentliche Verzerrung der Studienergebnisse zu vermeiden. In den verbleibenden sechs deutschen Venenzentren, welche die Studienanforderungen erfüllten, wurden insgesamt 405 konsekutive Patientinnen und Patienten (519 Beine) in die Studie eingeschlossen. Das mittlere Alter betrug 47,73 ± 10,05 Jahre. Es handelte sich um 258 Frauen (63,70 %) und 146 Männer (36,05 %). Der mittlere präoperative Durchmesser der VSM, gemessen mittels Duplexsonographie auf Höhe der SFJ, betrug 9,59 ± 2,99 mm. Bei 87,86 % aller Patienten reichte der Reflux bis zur Wade oder zum Knöchel, entsprechend den Hach‐Stadien III und IV. Zudem wiesen 95 % aller Patienten eine CEAP‐Klassifikation ≥ C2 auf. Weitere Basisdaten sind in Tabelle 1 dargestellt.

**TABELLE 1:** Demografische, klinische und therapiebezogene Basisdaten.

Anzahl	n = 405
Mittleres Alter in Jahren (SD)	47,73 (± 10,05)
Männlich (%)	146 (36,05)
Weiblich (%)	258 (63,70)
BMI (kg/m^2^)(SD)	27,39 (± 5,81)
Behandelte Extremitäten	n = 519
Rechtes Bein (%)	257 (49,52)
Linkes Bein (%)	262 (50,48)
Hach‐Klassifikation	
Stadium I (%)	0 (0)
Stadium II (%)	63 (12,14)
Stadium III (%)	287 (55,30)
Stadium IV (%)	169 (32,56)
CEAP‐Klassifikation	
C 0 (%)	0 (0)
C 1 (%)	23 (4,43)
C 2 (%)	363 (69,94)
C 3 (%)	100 (19,27)
C 4 (%)	27 (5,20)
C 5 (%)	1 (0,19)
C 6 (%)	5 (0,96)

*Abk*.: BMI, *Body‐Mass‐Index*; CEAP, *Clinical‐Etiological‐Anatomical‐Pathophysiological*; SD, Standardabweichung

Daten als absolute Zahlen, Mittelwert ± SD oder Prozentwerte angegeben.

Die Gesamtkomplikationsrate nach der Operation betrug 5,39 % (n = 28). Davon wiesen 22 (4,24 %) Patientinnen und Patienten eine Hypästhesien auf (zwei in der Leiste, 20 an den Inzisionsstellen der Mini‐Phlebektomien), vier (0,77 %) entwickelten Hämatome, die chirurgisch ausgeräumt werden mussten (zwei in der Leiste, zwei am Unterschenkel), und zwei (0,39 %) wiesen Lymphozelen auf, die konservativ behandelt wurden (eine in der Leiste, eine am Oberschenkel).

Am ersten postoperativen Tag erfolgte routinemäßig eine Duplexsonographie. Dabei zeigte sich kein residueller Stumpf und keine tiefe Venenthrombose. In vier (0,77 %) Fällen wurde eine oberflächliche Thrombophlebitis von Seitenästen im Oberschenkelbereich festgestellt, in drei (0,58 %) Fällen im Unterschenkelbereich. Ein venöser Reflux von über 0,5 s im oberflächlichen Venensystem wurde in zwölf (2,31 %) Fällen nachgewiesen – davon einer (0,19 %) am Oberschenkel und elf (2,12 %) am Unterschenkel. Behandlungsdaten, Komplikationsraten und postoperative sonographische Befunde sind in Tabelle 2 aufgeführt.

**TABELLE 2:** Behandlungsdaten, Komplikationen und postoperative sonographische Befunde.

Parameter	Gesamt (n = 519)
Ambulante Therapie (%)	67 (12,90)
Stationäre Behandlung (%)	452 (87,09)
Single‐Shot‐Heparingabe (%)	508 (97,88)
Fortgesetzte Heparintherapie (%)	15 (2,89)
Single‐Shot‐Antibiose (%)	273 (52,60)
Operative Komplikationen	
Motorische Nervenschädigung (%)	0
Sensorische Nervenschädigung (%)	22 (4,24)
Hämatom (operationspflichtig) (%)	4 (0,77)
Lymphozele (%)	2 (0,39)
Postoperative sonographische Befunde	
Tiefe Venenthrombose (%)	0
Sichtbarer VSM‐Stumpf (%)	0
Thrombophlebitis am Oberschenkel (%)	4 (0,77)
Thrombophlebitis am Unterschenkel (%)	3 (0,58)
Venöser Reflux (Oberschenkel) (%)	1 (0,19)
Venöser Reflux (Unterschenkel) (%)	11 (2,12)

Daten als absolute Zahlen und Prozent angegeben.

Nach 5 Jahren standen 225 Patienten (287 Beine) zur Nachuntersuchung zur Verfügung. Dies entspricht einer Follow‐up‐Rate von 55,56 % (bezogen auf Beine: 55,30 %).

Bei 21 (7,32 %) der 287 behandelten Beine wurden klinisch manifeste Varizen festgestellt, sodass gemäß REVAS‐Protokoll eine detaillierte Analyse erfolgte. In keinem dieser Fälle bestand ein topografischer Zusammenhang mit der Leiste. Ein Bein (0,35 %) zeigte eine Crosse‐Insuffizienz ausgehend von pelvinen Venen, und in zwei Fällen (0,70 %) wurde eine Perforansinsuffizienz der Vena poplitea gefunden. Die Mehrheit der klinisch erkennbaren Varizenrezidive war auf Perforansinsuffizienzen des Oberschenkels oder der unteren Extremität zurückzuführen.

Die Ergebnisse der Duplexsonographie unterschieden sich von der REVAS‐Klassifikation, da dort jeder nachgewiesene Reflux berücksichtigt wurde – auch klinisch inapparenter. Ein Rezidivreflux der SFJ wurde in fünf Fällen (1,74 %) duplexsonographisch nachgewiesen. Lediglich ein Fall (0,35 %) zeigte einen hämodynamisch relevanten Venendurchmesser von 7 mm. Bei den übrigen vier Fällen lag der Durchmesser der insuffizienten Leistenvenen unter 3,5 mm, weshalb sie als nicht hämodynamisch relevant eingestuft wurden. In 22 Beinen (7,67 %) wurde ein Reflux im Oberschenkelbereich festgestellt, mit einem mittleren maximalen Durchmesser von 3,18 (SD 1,67 mm), sowie 28 insuffiziente oberflächliche Venen der unteren Extremität mit einem mittleren Durchmesser von 2,80 (Standardabweichung; SD 1,43 mm). Die Fünf‐Jahres‐Ergebnisse sind in Tabelle 3 dargestellt.

**TABELLE 3:** Fünfjahresergebnisse.

Parameter	
Follow‐up‐Patienten (%) (initial n = 405)	225 (55,56)
Follow‐up‐Beine (%) (initial n = 519)	287 (55,30)
Klinisch manifeste Varikosis (REVAS) ( %)	21 (7,32)
Leistenvarikosis	0
Oberschenkelvarikosis (%)	1 (0,35)
Oberschenkel‐ und Unterschenkelvarikosis (%)	10 (3,48)
Unterschenkelvarikosis (%)	8 (2,79)
Popliteale Varikosis (%)	2 (0,70)
Sonographische Befunde	
Reflux an der SFJ (%)	5 (1,74)
Summe des venösen Durchmessers in der Leiste in mm (SD)	4,00 (± 1,70)
Reflux am Oberschenkel (%)	22 (7,67)
Maximaler Venendurchmesser (Oberschenkel) in mm (SD)	3,18 (± 1,67)
Reflux am Unterschenkel (%)	28 (9,76)
Maximaler Venendurchmesser (Unterschenkel) in mm (SD)	2,80 (± 1,43)
CEAP‐Klassifikation	
C 0 (%)	85 (29,62)
C 1 (%)	133 (46,34)
C 2 (%)	60 (20,91)
C3 (%)	9 (3,14)
C 4 (%)	0
C 5 (%)	0
C 6 (%)	0

*Abk*.: CEAP, *Clinical‐Etiological‐Anatomical‐Pathophysiological*; REVAS, *Recurrent varices after surgery*; SD, Standardabweichung; SFJ, *saphenofemoral junction*

Daten als absolute Zahlen, Mittelwert ± Standardabweichung (SD) oder Prozent dargestellt.

Der Student's t‐Test zeigte im Vergleich zum präoperativen Befund nach 5 Jahren eine signifikante Verbesserung des C‐Stadiums der CEAP‐Klassifikation (p < 0,001). Die Verteilung der CEAP‐C‐Stadien prä‐ und postoperativ nach 5 Jahren ist in Abbildung 1 dargestellt.

**ABBILDUNG 1:**
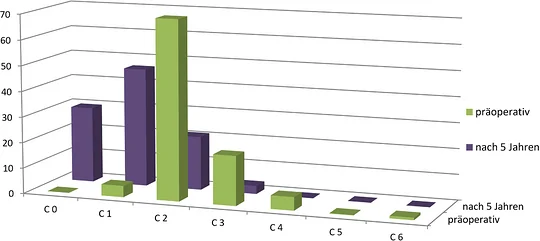
CEAP‐Klassifizierung: prozentuale Verteilung der C‐Stadien vor der Operation und nach 5 Jahren.

## DISKUSSION

In dieser prospektiven multizentrischen Studie lag die duplexsonographische Rezidivrate im Bereich der SFJ nach standardisierter Crossektomie nach 5 Jahren bei 1,74 %. Nur in einem Fall (0,35 %) war die Insuffizienz hämodynamisch relevant mit einem kumulierten Venendurchmesser von mehr als 5 mm. Obwohl der Schwerpunkt dieser Untersuchung auf Leistenrezidiven lag, wurden nach 5 Jahren klinische Rezidive in 7,32 % der Fälle festgestellt – und damit geringer als erwartet. Diese Ergebnisse stehen im Gegensatz zu früher publizierten Studien, da sie eine niedrigere Rezidivrate in der Leiste zeigen. So berichteten Allegra et al. in einer prospektiven Studie nach *Flush*‐Ligation der VSM über eine Rezidivrate an der SFJ von 13 % (109 von 862 Fällen), nachgewiesen mittels Duplexsonographie 5 Jahre nach der Erstoperation.^15^ Andere prospektive Studien wiesen Rezidivraten an der SFJ von 11,7 % nach 6 Jahren beziehungsweise 7 % nach 5 Jahren auf.^13^, ^14^ Dabei ist zu berücksichtigen, dass die in den genannten Arbeiten verwendeten Operationstechniken nicht im Detail beschrieben wurden und zum Teil heterogene Patientenkollektive eingeschlossen waren. Ein direkter Vergleich mit den in unserer Studie erhobenen Daten ist daher nur eingeschränkt möglich. Die ausführlichste Beschreibung der Operationstechnik findet sich in der sogenannten RELACS‐Studie von Rass et al.^16^ In dieser Studie bestand die Crossektomie in der Durchtrennung aller Seitenäste im Bereich der Leiste, der *Flush*‐Ligatur der SFJ mit einer nicht‐resorbierbaren Ethibond 0‐0‐Naht (Ethicon, New Brunswick, New Jersey) sowie einer neorefluxprotektiven, invaginierenden, fortlaufenden Prolene‐Naht 4‐0 (Ethicon) zum Stumpfverschluss, gefolgt von einem invaginierenden *Stripping* der VSM bis knapp unterhalb des Knies. Trotz dieser Technik berichteten die Autoren eine höhere Duplex‐Rezidivrate an der SFJ nach HL/S von 5 % nach 5 Jahren im Vergleich zu den Ergebnissen der vorliegenden Studie.

Möglicherweise lässt sich die niedrigere Rate an Leistenrezidiven in unserer Studie durch die spezifische operative Technik der HL/S erklären. Zum einen wurde die Vena femoralis communis in einem kleinen Bereich um die SFJ freigelegt, und additiv die direkt einmündenden Seitenäste dargestellt und ligiert. Anatomische Untersuchungen konnten bestätigen, dass in 57 % der Fälle mindestens eine Vene direkt auf Höhe der SFJ in die Vena femoralis communis mündet.^25^ Darüber hinaus wurde in der vorliegenden Arbeit eine monofile, nicht‐resorbierbare Ligatur für den Stumpf der VSM verwendet, und die freiliegende Intima des Stumpfes wurde bipolar koaguliert, um eine neointimale Induktion zu vermeiden. Ein weiterer Unterschied zur RELACS‐Studie besteht darin, dass dort keine thermische Ablation durchgeführt wurde.^15^ Ob dies zu den verbesserten Ergebnissen in unserer Studie beigetragen hat, lässt sich nicht mit Sicherheit sagen. Es gibt jedoch Hinweise mit niedriger Evidenz, dass bei Verwendung nicht‐resorbierbarer Nähte die Rezidivrate an der SFJ geringer ist als bei resorbierbarem Nahtmaterial.^26^ Frings et al. berichteten über eine Zwei‐Drittel‐Reduktion der SFJ‐Rezidive nach Exklusion des VSM‐Stumpfes mittels einer übernähten, synthetischen, monofilen Naht,^27^ im Vergleich zu einem freiliegenden Stumpf mit einfacher Ligatur – Ergebnisse, die allerdings von anderen Autoren nicht bestätigt werden konnten.^28^ Ein weiterer möglicher Einflussfaktor ist die operative Erfahrung: Alle beteiligten Operateure waren in der atraumatischen Präparation der SFJ besonders geschult, mit dem Ziel einer minimalen Gewebetrauma‐ und Hämatombildung, um angiogene Stimuli zu vermeiden. Dies könnte ebenfalls zu den guten Ergebnissen beigetragen haben, da die Qualifikation der Chirurgen nachweislich Einfluss auf den Operationserfolg hat.^29^

Darüber hinaus gehen wir davon aus, dass der Verschluss der Fascia cribrosa ein weiterer entscheidender Faktor zur Vermeidung eines Leistenrezidivs nach HL/S der VSM ist. Der Verschluss der Fossa ovalis fungiert als anatomische Barriere, die das tiefe vom oberflächlichen Venensystem trennt. Dies stellt einen wesentlichen Unterschied zum RELACS‐Studienprotokoll dar, in dem kein Faszienverschluss oder andere Barriere‐Maßnahmen durchgeführt wurden.^16^ Das Konzept einer prothetischen oder anatomischen Barriere zur Vermeidung von Neovaskularisation wurde bereits vor 18 Jahren vorgeschlagen.^30^ De Maeseneer et al. konnten nachweisen, dass der Verschluss der Fascia cribrosa, mit oder ohne Verwendung eines prothetischen Materials, die Rezidivrate an der SFJ nach HL/S der VSM signifikant reduziert.^31^, ^32^ Auf Basis dieser Ergebnisse wurde der Faszienverschluss auch in der vorliegenden Studie als eine potenzielle Barriere gegen transfasziale Insuffizienzen integriert.

Zudem können in der Duplexsonographie detektierte Neorefluxe der SFJ in der Zukunft klinisch relevant werden, da rezidivierender Leistenreflux an der SFJ ein bekannter Surrogatparameter für die spätere Entwicklung klinischer Rezidive ist.^5^, ^16^

Daher sollten die veröffentlichten Daten kurz‐ und mittelfristiger Duplex‐Rezidivraten nach chirurgischen und endovaskulären Behandlungsverfahren sorgfältig bewertet werden, da sie Rückschlüsse auf das langfristige klinische Outcome zulassen könnten. In diesem Zusammenhang liegen derzeit die meisten Langzeitdaten für die endovenöse Laserablation (EVLA) vor. Beispielsweise zeigte sich in vergleichbaren Studien mit 5 oder mehr Jahren Nachbeobachtung, dass EVLA signifikant häufiger duplexsonographisch nachweisbare Rezidive an der SFJ aufwies.^13^, ^16^, ^33^ Unabhängig von den Duplexbefunden konnten Rass et al. und Gauw et al. zeigen, dass EVLA nach 5 Jahren signifikant mehr klinisch relevante Rezidive an der SFJ verursachte als HL/S.^14^, ^16^ Allerdings fanden sich in diesen Studien keine signifikanten Unterschiede in der Gesamt‐Rezidivrate zwischen beiden Gruppen. Nach den Daten der deutschen Leistenrezidiv‐Studie treten klinisch manifeste Leistenrezidive im Mittel erst nach sieben bis acht Jahren auf.^3^, ^4^ Dies deutet darauf hin, dass zukünftige Studien mit längeren Nachbeobachtungszeiten erforderlich sind, um die klinischen Ergebnisse verschiedener Therapieverfahren zuverlässig vergleichen zu können. Nach unserem Kenntnisstand liegt bislang nur eine Studie mit zehnjährigen Langzeitergebnissen zu diesem Thema vor.^34^ Darin wurden signifikant weniger klinisch manifeste Leistenrezidive nach HL/S im Vergleich zu EVLA beobachtet. Die Aussagekraft der Studie ist jedoch aufgrund methodischer Einschränkungen begrenzt.

Die geringe Leistenrezidivrate in der vorliegenden Studie nach technisch korrekter Crossektomie über 5 Jahre bestätigt das von den phlebologischen Pionieren Dodd und Nabatov formulierte Motto: „*No stump – no recurrence*.“^35^, ^36^

Diese Erkenntnis könnte auch die Grundlage dafür sein, dass endovenöse Therapieverfahren mit sogenannter „endovenöser Crossektomie“ eine ähnliche Rezidivreduktion erzielen können, wie eine kürzlich publizierte, randomisiert‐kontrollierte Studie zeigen konnte.^37^

Nicht zuletzt spielt auch die Patientenperspektive, mit Erwartungen und Präferenzen eine zentrale Rolle bei der Wahl der Therapie und der zukünftigen Versorgung. In einer Befragung von Shepard et al. zu den Behandlungspräferenzen von Varizenpatienten zeigte sich, dass mehr als die Hälfte der Befragten eine einmalige Behandlung einer wiederholten Therapie vorziehen.^38^ Maßnahmen zur Vermeidung von Rezidiven sind somit ein entscheidender Faktor bei der Therapieentscheidung und verdienen besondere Berücksichtigung in der klinischen Praxis.

### Studienlimitationen

Dennoch weist die vorliegende Studie mehrere Limitationen auf. Eine wesentliche Einschränkung besteht darin, dass sechs der ursprünglich zwölf Venenzentren aufgrund unzureichender Nachuntersuchungsraten nicht in die Auswertung der Langzeitdaten einbezogen werden konnten. Hierfür gibt es verschiedene Gründe. Beispielsweise schieden im Verlauf der Studie einige der ursprünglich beteiligten klinischen Untersucher aus oder wechselten die Einrichtung. Diese eingeschränkten *Follow‐up*‐Daten verdeutlichen die erschwerten Studienbedingungen für multizentrische Untersuchungen bei Varikose‐Erkrankungen in Deutschland. Ein Grund hierfür ist, dass die Venentherapie in Deutschland überwiegend in nichtuniversitären ambulanten Einrichtungen erfolgt. Diese Zentren unterliegen inzwischen starken wirtschaftlichen und personellen Einschränkungen, sodass sich die äußeren Rahmenbedingungen für die einzelnen Studienzentren im Laufe der Zeit verändert haben. Dies könnte die begrenzte Zahl der Zentren, die in die Fünf‐Jahres‐Auswertung einbezogen werden konnten, erklären. Trotzdem konnten insgesamt 287 Beine nach 5 Jahren eingeschlossen werden, was einer Nachuntersuchungsrate von 55,56 % entspricht. Es kann jedoch nicht ausgeschlossen werden, dass die reduzierte Follow‐up‐Rate zu einer Verzerrung der Ergebnisse geführt hat. Im Vergleich zu anderen Studien zu diesem Thema konnten wir jedoch höhere Fallzahlen und Nachuntersuchungsraten erreichen,^14^, ^15^, ^16^, ^17^, ^19^ weshalb wir glauben, dass die Ergebnisse unserer Untersuchung einen wesentlichen Beitrag zur wissenschaftlichen Diskussion leisten können. Eine weitere Limitation unserer Studie ist das Fehlen eines vergleichenden Studiendesigns beziehungsweise einer Kontrollgruppe. Die Studie war jedoch von Beginn an als Beobachtungsstudie konzipiert, um die Ergebnisse einer standardisierten HL/S‐Technik mit besonderem Augenmerk auf Leistenrezidive im Langzeit‐Follow‐up zu untersuchen, da viele Publikationen historische HL/S‐Ergebnisse als Vergleichsgruppe heranziehen, ohne die konkrete Operationstechnik zu definieren. Das Fehlen einer Kontrollgruppe oder eines direkten Vergleichsansatzes schränkt daher die Aussagekraft der Ergebnisse nicht ein und verhindert die fundierte Interpretation der Resultate nicht.

### Schlussfolgerungen

In der vorliegenden Studie konnte gezeigt werden, dass eine standardisierte High‐Ligation‐und‐Stripping‐Technik nach 5 Jahren eine Leistenrezidivrate von lediglich 1,74 % aufweist. Diese Ergebnisse unterstreichen die Wirksamkeit der *High‐Ligation* bei der Vermeidung von Leistenrezidiven. Unklar bleibt, welcher spezifische Bestandteil der angewandten Operationstechnik entscheidend für dieses gute Ergebnis ist. In jedem Fall sind vergleichende Studien mit langfristigem *Follow‐up* erforderlich, um die standardisierte Crossektomie im Hinblick auf die Leistenrezidivrate mit anderen Therapieverfahren zu vergleichen und ihre Langzeiteffektivität zu bewerten.

## DANKSAGUNG

Open access Veröffentlichung ermöglicht und organisiert durch Projekt DEAL.

## FINANZIERUNG

Die Studie wurde von der Arbeitsgruppe Venenchirurgie (VOP‐AG) der Deutschen Gesellschaft für Phlebologie (DGP) initiiert und finanziell unterstützt. Die finanzielle Unterstützung hatte keinen Einfluss auf die Analyse und Interpretation der Daten.

## INTERESSENKONFLIKT

Keiner.
